# Ultrasound Guided Peripheral Nerve Block Workshop: How to Take Your Residents from Zero to Hero

**DOI:** 10.21980/J8.52156

**Published:** 2025-10-31

**Authors:** Ashley Foreman, Jacqueline Sippel, Emily Ollmann, Nicole Dorinzi

**Affiliations:** * West Virginia University School of Medicine, Department of Emergency Medicine, Morgantown, WV

## Abstract

**Audience:**

Emergency medicine residents of all years of training

**Introduction:**

Ultrasound guided peripheral nerve blocks (USGPNBs) provide adequate analgesic management by targeting specific nerves while limiting systemic effects.^1^,^2^ Utilizing this form of analgesia can decrease the use of procedural sedation and/or systemic pain management, which can pose inherent risks such as need for continuous monitoring and airway compromise.^1^ Nerve blocks require both training and specialized equipment to perform. This workshop aimed to evaluate resident comfort and familiarity with these blocks before and after completing specialized training sessions focusing on serratus anterior, posterior tibial, median, ulnar, and radial USGPNB. Ultrasound training for most residents is a longitudinal experience that often includes dedicated ultrasound scanning shifts and encouragement to include ultrasound into daily practice, however, the utility of ultrasound workshops in teaching nerve blocks has up until this point been explored very little and therefore under-utilized.

**Educational Objectives:**

After completing this small group workshop, the resident should be able to: 1) recognize the indications for the serratus anterior plane block, the posterior tibial block and the ulnar, median, and radial nerve blocks and the anatomical locations that would benefit from these blocks, 2) identify proper probe selection and placement, in addition to patient positioning, in order to perform these blocks, as well as anesthetic choice and dosing, 3) demonstrate knowledge of anatomical landmarks and areas to avoid evidenced by probe placement and positioning, 4) describe the steps to perform these nerve blocks, and 5) demonstrate knowledge of contraindications to these blocks as well as potential complications of these procedures and how to mitigate them.

**Educational Methods:**

This workshop was held in a small group format that was set up in multiple stations. Each station started with a short didactic presentation followed by USGPNB simulation on standardized patients.

**Research Methods:**

Pre-workshop surveys were conducted via Qualtrics to assess familiarity with the indications and mechanics for conducting UGPNBs. Following the workshop, the survey was repeated as a post-workshop assessment, where residents were given the opportunity to assess their comfort with the indications for these blocks and their confidence in the steps taken to perform them. They also gave feedback on the workshop design and content.

**Results:**

Overall, residents reported more comfort with these blocks based on survey results. Pre-workshop survey responses contained widespread levels of comfort in all aspects of these blocks including knowledge pertaining to indications, probe selection, anatomical regions that would benefit from anesthetic, anatomical landmarks, areas to avoid, anesthetic choice, contraindications, and complications and their respective management. Following the workshop, all participants voiced increased knowledge and confidence, answering that they somewhat or strongly agreed with comfortability in all of the above areas 93–100% of the time.

**Discussion:**

Small group workshops that utilize standardized patients (SPs) and concise didactic presentations are effective ways to teach emergency medicine residents how to confidently perform UGPNBs.

**Topics:**

Nerve block, ultrasound, small group, workshop, resident, local anesthesia.

## USER GUIDE

**Table:** 

List of Resources:	
Abstract	66
User Guide	68
Small Groups Learning Materials	71
Appendix A: Small Group Application Exercise	71
Appendix B: Nerve Block PowerPoint	80
Appendix C: Nerve Block Surveys	81

**Learner Audience:**

Interns, Junior Residents, Senior Residents

**Time Required for Implementation:**

1 hour per iteration of small group of residents divided into three 20-minute stations dedicated to each respective block. No significant prep work for SPs was needed. Instructors needed approximately one hour for individual prep time reviewing nerve block methodology from sources listed below.

**Recommended Number of Learners Per Instructor:**

3–5

**Topics:**

Nerve block, ultrasound, small group, workshop, resident, local anesthesia

**Objectives:**

After completing this small group workshop, the resident should be able to:

1. Recognize the indications for the serratus anterior plane block, the posterior tibial block and the ulnar, median, and radial nerve blocks and the anatomical locations that would benefit from these blocks.
2. Identify proper probe selection and placement in order to perform these blocks, as well as anesthetic choice and dosing.
3. Demonstrate knowledge of anatomical landmarks and areas to avoid evidenced by probe placement and positioning.
4. Describe the steps to perform these nerve blocks.
5. Demonstrate knowledge of contraindications to these blocks as well as potential complications of these procedures and how to mitigate them.

### Linked objectives and methods

Ultrasound-guided nerve blocks (USGPNB) target specific nerves via ultrasound guidance in order to provide regional anesthesia while limiting systemic effects. This reduces the need for procedural sedation while providing safe and adequate anesthesia during painful procedures. This method of analgesia decreases complications associated with procedural sedation such as the need for airway monitoring and post sedation recovery. This can, therefore, reduce overall time spent in the ED leading to faster bed turn over, increased throughput, and increased patient satisfaction.^1^ USGPNBs are fast, effective, and safe while minimizing patient discomfort and optimizing patient's satisfaction by adequately controlling patient's pain. USGPNBs offer several advantages including:

1. Direct visualization of the nerves and surrounding anatomical structures (ie, vessels, muscles, bone, fascia, tendon) which assists in structure identification in regard to individual anatomical variation.^6^
2. Real-time control of needle advancement which allows for reduced number of attempts, shortens the time for effective block completion, and lowers the risk of complications (ie, vascular puncture, neurapraxia, damage to surrounding structures such as pneumothoraxes).^6^
3. Allows for assessment of anesthetic spread and allows for immediate supplementary injections in case of insufficient spread which can improve block effectiveness, shorten block completion time, and provides prolonged duration of block effectiveness. This in turn allows for overall reduction in dose needed for the anesthetic and lowers the risk of anesthetic overdose.^6^

However, USGPNBs require both personal skills and high-quality ultrasound equipment. Inadequate emergency physician training in use of ultrasound, lack of physician comfort level with the use of these techniques, and lack of specialized equipment all lead to decreased implementation of this procedure in the ED.^1^

Potential complications of anesthetics include vascular puncture and intravascular injection, peripheral nerve injury, pneumothorax^2^, infection, hematoma, and local anesthetic systemic toxicity. Local anesthetic systemic toxicity (LAST), which occurs due to either intravascular injection or systemic absorption of large volumes of anesthetics, causes central nervous system and cardiovascular effects, with initial symptoms potentially being delayed up to one hour due to absorption time.^7^ Specifically, symptoms of LAST typically arise within the first minute of injection, initially presenting with tachycardia and hypertension before turning into bradycardia, hypotension, dysrhythmia, and in some cases asystole, with some reports of seizure. Mainstays of treatment largely include symptomatic management with benzodiazepines for seizures, as well as advanced cardiac life support. Twenty percent lipid emulsion can be used to reverse the effects of LAST, with a 1.5 mL/kg bolus followed by an infusion at a rate of 0.25 mg/kg/min.^8^ Therefore, ultrasound guidance can be used to potentially decrease these serious complications.

The goal of this workshop was to introduce residents to five USGPNB that would be beneficial in everyday practice for common complaints presenting to the ED. We hypothesized that by educating residents on USGPBN via active learning through use of SPs that residents would have increased awareness and confidence in performing nerve blocks in the future. After reviewing the PowerPoint slides (objectives 1, 2, 5), learners were given the opportunity to practice their skills on SPs (objectives 3 and 4) under the guidance of faculty and senior resident preceptors. Small group format was chosen as the ideal teaching environment so that residents got more personalized teaching and had the ability to practice identifying the anatomy in real time on SPs individually so that they feel comfortable in the future identifying these structures and performing these USGPNB on patients in their practice.

### Recommended pre-reading for facilitator

Each USGPNB taught in this paper can be reviewed on the New York School of Regional Anesthesia (NYSORA) website:

- Lin J, Bendtsen TF, Lopez AM, Jalil H. Ultrasound-guided blocks at the elbow. New York School of Regional Anesthesia (NYSORA). Published 2024. https://www.nysora.com/topics/regional-anesthesia-for-specific-surgical-procedures/upper-extremity-regional-anesthesia-for-specific-surgical-procedures/anesthesia-and-analgesia-for-hand-procedures/ultrasound-guided-blocks-elbow/
- Vandepitte C, Lopez AM, Van Boxstael S, Jalil H. Ultrasound-guided ankle nerve block. New York School of Regional Anesthesia (NYSORA). Published 2024. https://www.nysora.com/topics/regional-anesthesia-for-specific-surgical-procedures/lower-extremity-regional-anesthesia-for-specific-surgical-procedures/foot-and-anckle/ultrasound-guided-ankle-block/
- Blanco R, Barrington MJ. Pectoralis and serratus plane nerve blocks. New York School of Regional Anesthesia (NYSORA). Published 2024. https://www.nysora.com/topics/regional-anesthesia-for-specific-surgical-procedures/thorax/pectoralis-serratus-plane-blocks/

### Small group application exercise (sGAE)

See the following attached materials for this small group exercise

- Appendix A: sGAE
- Appendix B: Nerve Block PowerPoint
- Appendix C: Nerve Block Surveys

### Results and Tips for Successful Implementation

The residents included in this study included PGY-1, PGY-2, and PGY-3 physicians with varying levels of experience with ultrasound and regional anesthesia. At the beginning of each station of the workshop, preceptors took the opportunity to review the indications, contraindications, methodology and anesthetic choice with typical and maximum dosing, as well as the potential complications and treatments for the aforementioned blocks.

This workshop took place in a simulation center after an already-scheduled simulation case had been completed by the group of residents. For the purpose of this study, the workshop was completed over four sessions because the totality of the participating residency program is split into four groups of varying PGY training levels that rotate each week in participating in simulation cases.

Prior to completion of the workshop, residents were given a Qualtrics link to a pre-survey that asked them to self-assess their familiarity and comfort with these five peripheral nerve blocks, specifically assessing the statements:

1. I am familiar with indications for forearm blocks, serratus anterior plane blocks, and posterior tibial nerve blocks.
2. I am familiar with the anatomical regions that would benefit from anesthetic injection during these blocks.
3. I am comfortable with probe selection while performing these blocks.
4. I am comfortable identifying anatomical landmarks pertinent to performing these blocks.
5. I am aware of the anatomical areas to avoid while performing these blocks.
6. I am familiar with the contraindications to performing these blocks.
7. I am comfortable choosing an injectable anesthetic for these blocks.
8. I am aware of the potential complications of these blocks and how to manage them.

They were asked to rate their answers on a scale from strongly disagree, disagree, neither agree nor disagree, agree, or strongly agree.

After the completion of all three stations, the residents were given a post-survey with the same questions to reassess their level of confidence in these nerve blocks with the addition of a free-text box in order to give personalized feedback on the content and design of the workshop as well as an opportunity to voice any lingering questions or hesitations about utilizing these procedures in their practice.

Overall, responses to the workshop on the surveys were promising. In pre-workshop survey responses, only 31% somewhat agreed or strongly agreed with the statement that they are comfortable with the indications of these blocks, 25% answered the same for comfort in knowing anatomical landmarks that would benefit from these blocks, and 62% answered similarly for comfort in probe selection. Only 13% chose the answers above when asked if they knew anatomical landmarks pertinent to successfully performing the blocks. Nineteen percent somewhat agreed or strongly agreed that they knew the areas to avoid while performing the blocks, 50% gave comparable answers to understanding contraindications, 50% answered the same for comfort in choosing an anesthetic, and 44% chose the options somewhat agreed or strongly agreed for understanding the potential complications of the blocks and how to manage them. This is in comparison to post-workshop responses of somewhat agree and strongly agree which were answered 100% of the time in all categories except for knowledge of anatomical landmarks that would benefit from the block, for which the above responses were chosen 93% of the time. Post-workshop surveys also included the option to expand upon any lingering concerns about implementing these blocks independently in the community. The few responses we did receive mentioned concerns about having time and the supplies necessary, while one person voiced concern about using an actual needle, which is a limiting factor while using standardized patients.

Given these responses, it seems that overall small workshops are effective ways to teach USGPNBs to resident physicians. In order to improve upon certain limitations of this study, in the future it may be worthwhile to incorporate a cadaver lab so that residents have the opportunity to approach the blocks with actual needles and set up the lines and equipment needed to perform the blocks, including diluting anesthetics for the plane block, so that they may be more confident and efficient in their own practice in the future.

## Supplementary Information



## Figures and Tables

**Figure:**
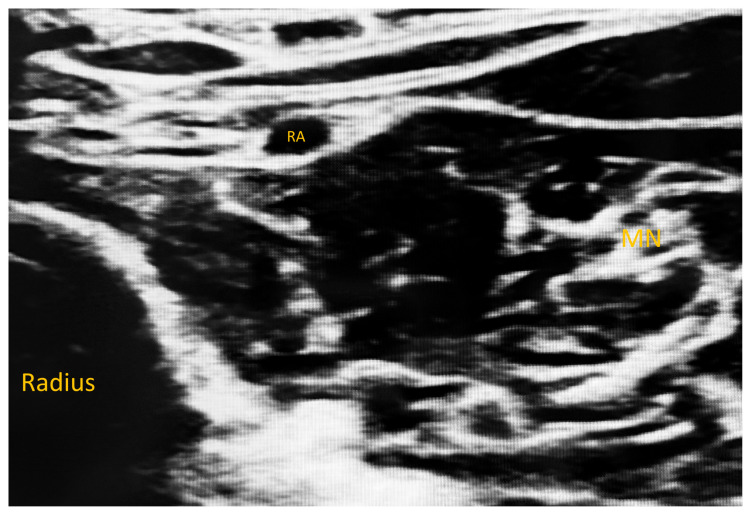
Ultrasound-guided median nerve block. Author’s own image.

**Figure:**
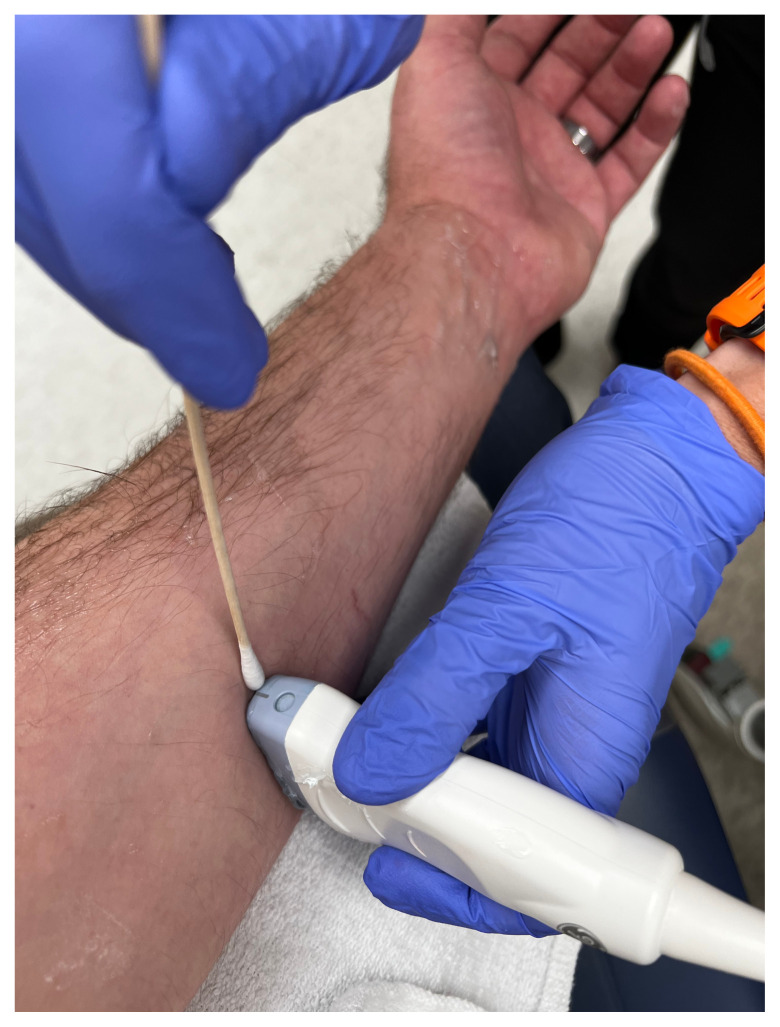
Ultrasound approach for median nerve block. Author’s own image.

**Figure:**
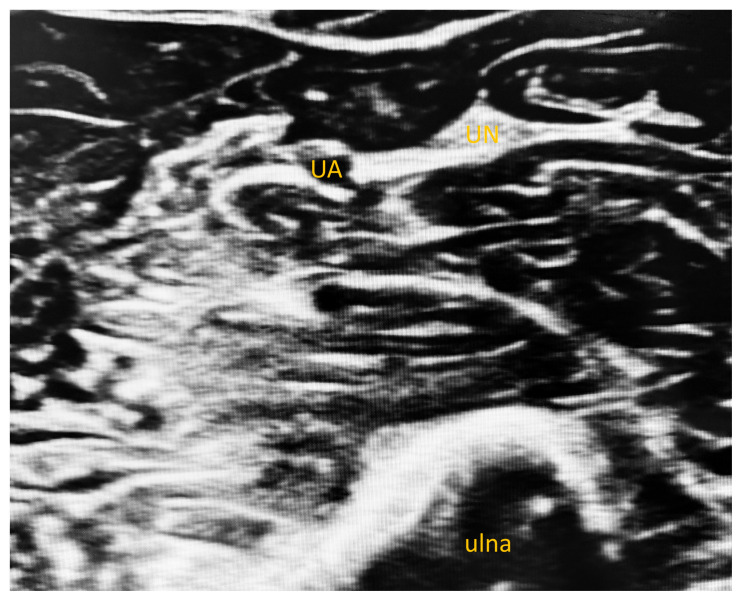
Ultrasound-guided ulnar nerve block. Author's own image.

**Figure:**
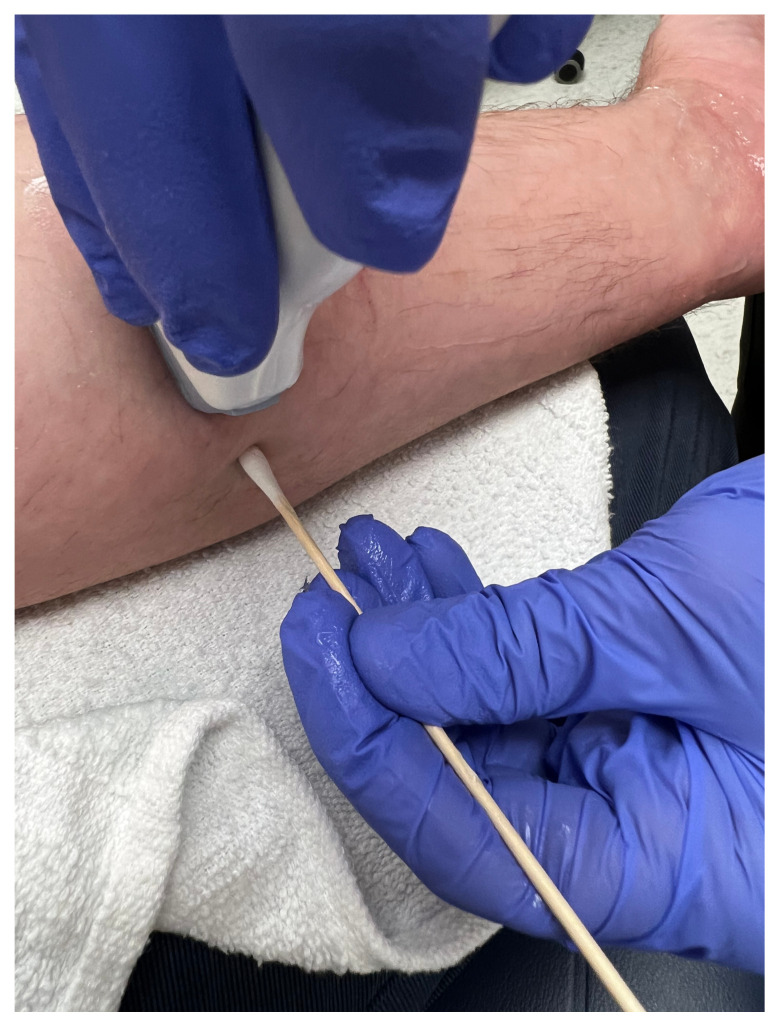
Ultrasound approach for ulnar nerve block. Author’s own image.

**Figure:**
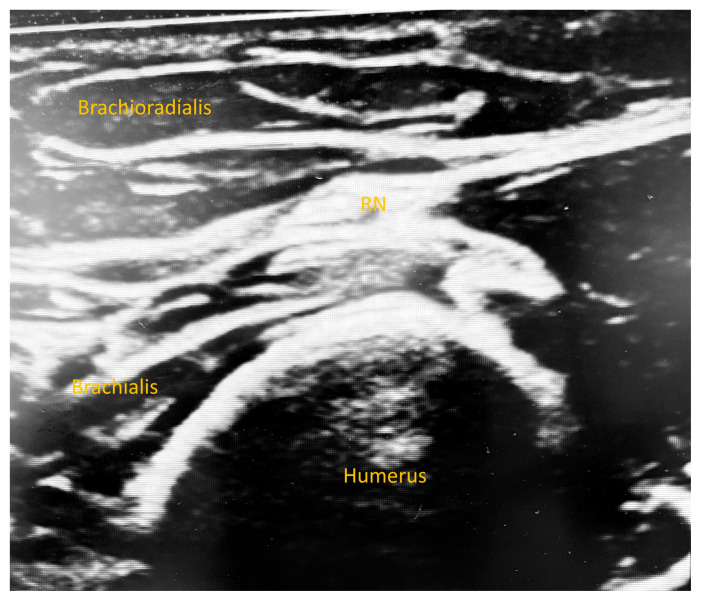
Ultrasound-guided radial nerve block. Author's own image.

**Figure:**
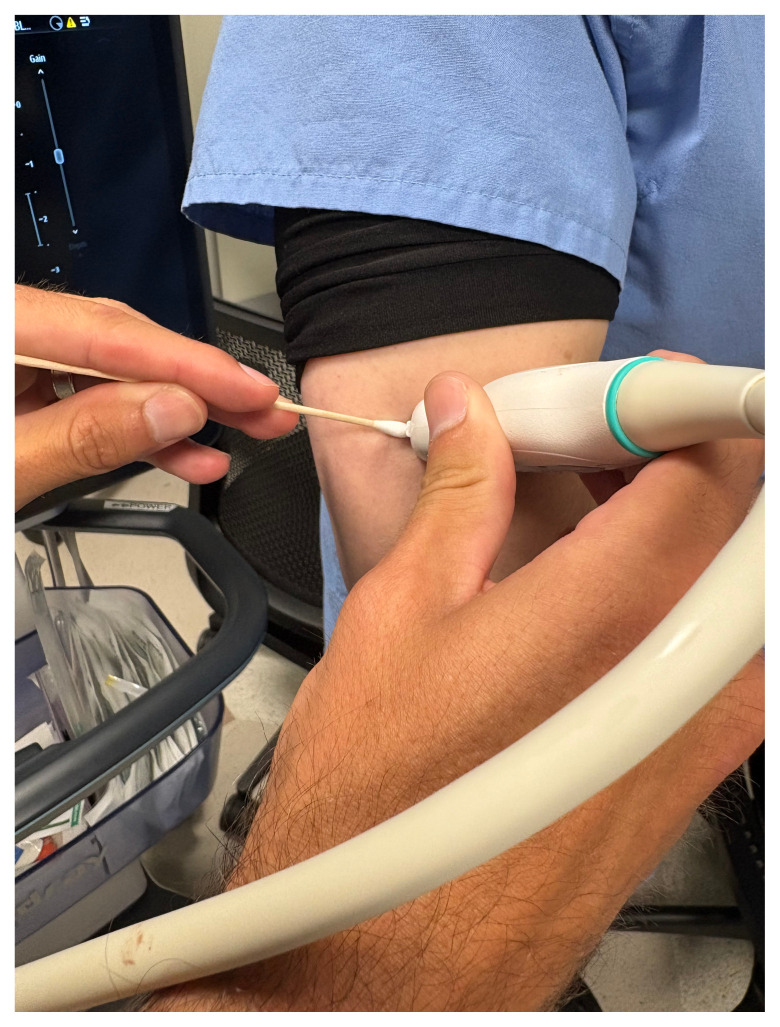
Ultrasound approach for radial nerve block. Author’s own image.

**Figure:**
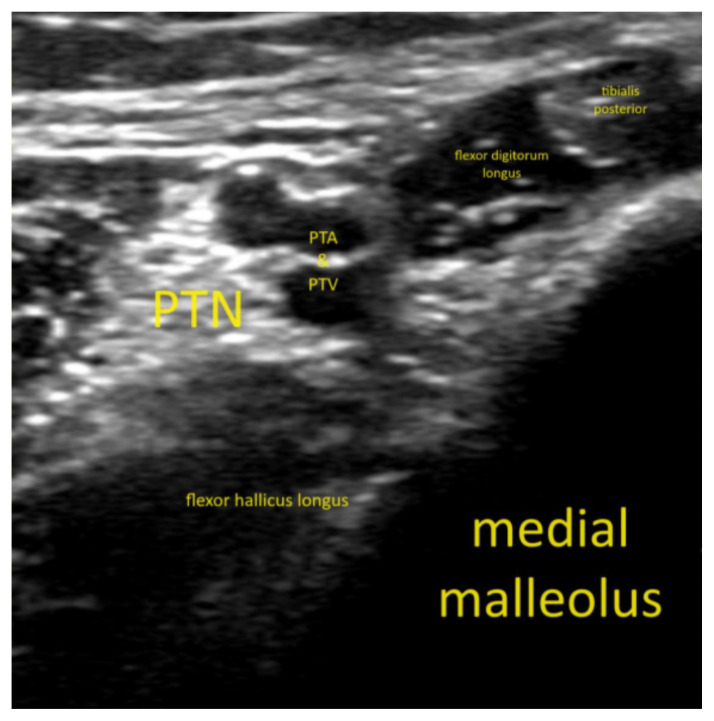
Ultrasound-guided posterior tibial nerve block. Author’s own image.

**Figure:**
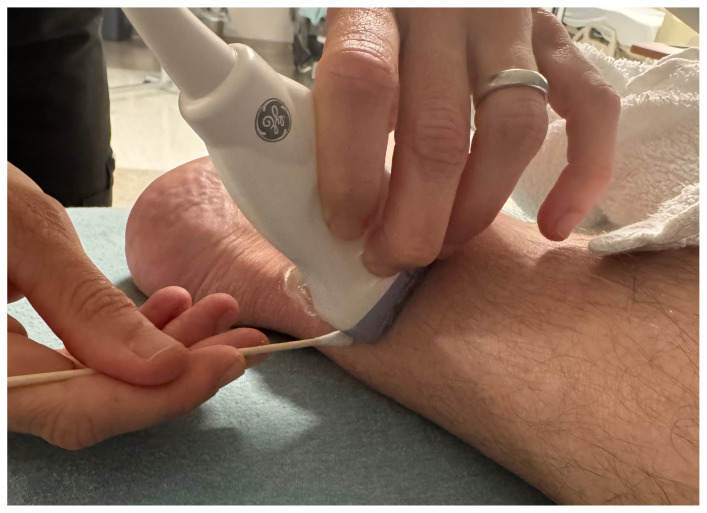
Ultrasound approach for posterior tibial nerve block. Author’s own image.

**Figure:**
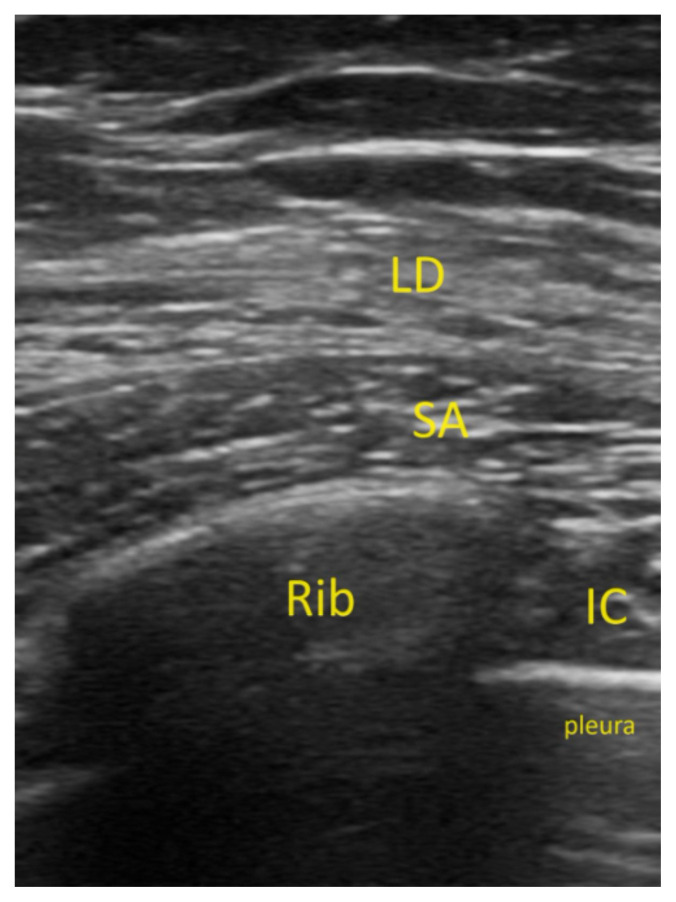
Ultrasound-guided serratus anterior plane block. Author’s own image.

**Figure:**
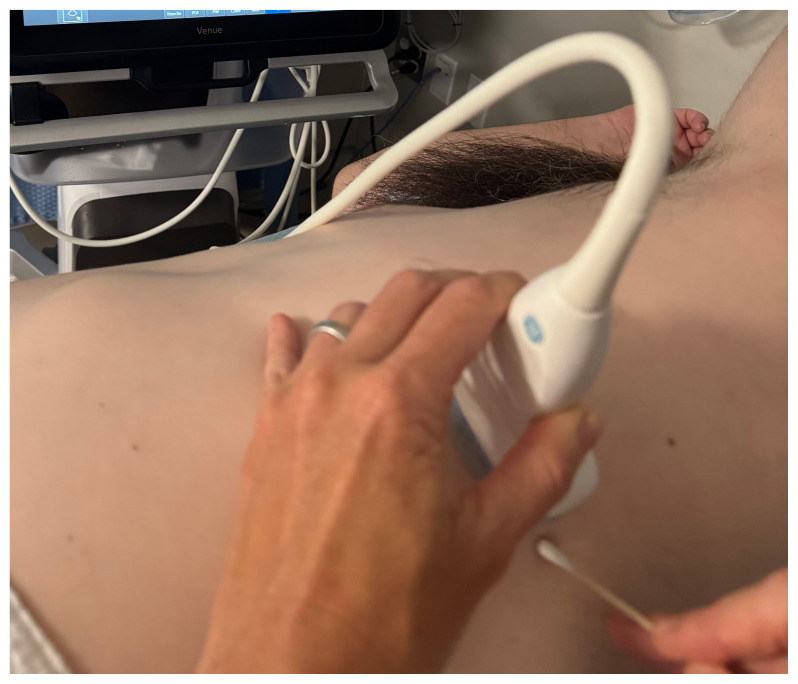
Ultrasound approach for serratus anterior plane block. Author’s own image.

**Figure:**
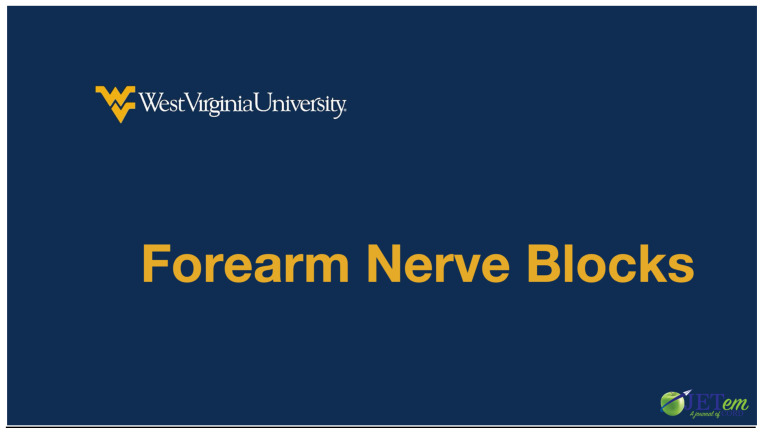
Please see associated PowerPoint file

## SMALL GROUPS LEARNING MATERIALS

### Appendix A Small Group Application Exercise

In this workshop, we taught several topics and skills including indications for performing five separate peripheral nerve blocks as well as how to position the bodies of SPs, which ultrasound (US) probe to choose, and how to identify different anatomical landmarks by US in order to successfully perform these blocks.

Prior to the workshop, facilitators reviewed various literature and video manuals on the performance of these five blocks:

- Serratus Anterior Plane Block
- Posterior Tibial Nerve Block
- Median Nerve Block
- Ulnar Nerve Block
- Radial Nerve Block

**Stations:**

Station set-up included:

1. One SP per station
2. One ultrasound per station
3. A computer with preloaded reference materials^7^/presentation loaded

Each station was set up in the SIM center with three SP’s posed on three exam tables. An ultrasound machine and computer were designated for each station. The computer at each station was preloaded with a PowerPoint presentation for each of the facilitator’s targeted block(s). Included in each PowerPoint were images of ultrasound-guided landmarks for each block captured by the authors of this paper. Please see Appendix A for these presentations. Prior to each workshop, a survey was given to each participant to gauge their familiarity with USGPNB (serratus anterior, posterior tibial, median, ulnar, and radial nerve blocks) including their indications and contraindications, as well as basic methodology including probe selection and landmark identification.

The residents self-selected a station to start at and were cycled through three separate stations over which these blocks were taught. The targeted blocks were divided into three stations: serratus anterior plane block, posterior tibial nerve block, and forearm nerve blocks (median, ulnar, and radial nerve).

Preceptors included the three resident authors of this study and a fellowship-trained ultrasound faculty member, who was floating at all three stations. The resident authors were responsible for the didactic presentation and modeling ultrasound technique for the block, and the faculty member oversaw teachings at all three stations while answering questions and expounding upon methodology as needed.

The residents spent a total time of approximately 10–20 minutes learning each block and cycled through the stations until they had participated in all three. Preceptors at each station started the presentation with a short didactic portion using a preloaded PowerPoint presentation on a computer in the room. The PowerPoint presentation included the indications, contraindications, methodology, potential complications, and reference pictures on how to successfully approach these blocks.

Following this, residents were given the opportunity to watch the preceptors model appropriate ultrasound techniques to identify landmarks and approach the various nerves on a SP. Facilitators positioned SPs in the appropriate positions (supinated forearm, flexed elbow for the forearm blocks), (lateral decubitus for serratus anterior plane block), (lateral decubitus or supine with affected leg in frog-leg position for posterior tibial nerve block). A linear transducer was selected and positioned on the SP until anatomic landmarks were identified. A cotton swab was used as a surrogate for a needle and advanced along the plane of the probe. The pressure of the swab was used to model the tract of the hypothetical needle as it approaches the nerve. Images below demonstrate these techniques.

**Forearm Blocks:**

Median Nerve:

Ulnar Nerve:

Radial Nerve:

Posterior Tibial Nerve:

Serratus Anterior Plane Block:

Residents were then given an opportunity to practice themselves while questions were answered and adjustments to technique were given in real time. Residents were given cotton-tipped probes to practice needle approach on the standardized patient. Residents were allowed to self-guide the length of each station, completing them when they felt confident in what they had learned.

Following this, participants were educated on the above aspects of these blocks by listening to a PowerPoint presentation and watching preceptors demonstrate how to perform these blocks on SPs before ultimately practicing themselves by posing SPs in optimal positions, using the ultrasound machine to find landmarks, and using blunt probes to practice needle approach in or out of plane. No markings were performed on SPs, but during this time, they were given the opportunity to ask questions and voice concerns as they arose.

At the end of the workshop, participants were given the same survey with the exception of an additional open-ended question on any concerns or hesitations they might still have. Their answers were recorded anonymously and compared to their anonymous pre-workshop responses to assess the effectiveness of the workshop.

### Appendix C Nerve Block Surveys

Ultrasound-Guided Serratus Anterior Plane Block Pre-Workshop Questionnaire

1. I am familiar with the indications of a serratus anterior plane block.
  **Table:**
  Strongly Disagree	Disagree	Neutral	Agree	Strongly Agree
  1	2	3	4	5
2. I am familiar with the anatomical regions that would benefit from anesthetic injection during a serratus anterior plane block.
  **Table:**
  Strongly Disagree	Disagree	Neutral	Agree	Strongly Agree
  1	2	3	4	5
3. I am comfortable with probe selection while performing this plane block.
  **Table:**
  Strongly Disagree	Disagree	Neutral	Agree	Strongly Agree
  1	2	3	4	5
4. I am comfortable identifying the anatomical landmarks pertinent to performing this block.
  **Table:**
  Strongly Disagree	Disagree	Neutral	Agree	Strongly Agree
  1	2	3	4	5
5. I am aware of the anatomical areas to avoid while performing this block.
  **Table:**
  Strongly Disagree	Disagree	Neutral	Agree	Strongly Agree
  1	2	3	4	5
6. I am familiar with the contraindications to performing this block.
  **Table:**
  Strongly Disagree	Disagree	Neutral	Agree	Strongly Agree
  1	2	3	4	5
7. I am comfortable choosing an injectable anesthetic for this block.
  **Table:**
  Strongly Disagree	Disagree	Neutral	Agree	Strongly Agree
  1	2	3	4	5
8. I am aware of the potential complications of this block and how to manage them.
  **Table:**
  Strongly Disagree	Disagree	Neutral	Agree	Strongly Agree
  1	2	3	4	5

Ultrasound-Guided Serratus Anterior Plane Block Post-Workshop Questionnaire

1. I am familiar with the indications of a serratus anterior plane block.
  **Table:**
  Strongly Disagree	Disagree	Neutral	Agree	Strongly Agree
  1	2	3	4	5
2. I am familiar with the anatomical regions that would benefit from anesthetic injection during a serratus anterior plane block.
  **Table:**
  Strongly Disagree	Disagree	Neutral	Agree	Strongly Agree
  1	2	3	4	5
3. I am comfortable with probe selection while performing this plane block.
  **Table:**
  Strongly Disagree	Disagree	Neutral	Agree	Strongly Agree
  1	2	3	4	5
4. I am comfortable identifying the anatomical landmarks pertinent to performing this block.
  **Table:**
  Strongly Disagree	Disagree	Neutral	Agree	Strongly Agree
  1	2	3	4	5
5. I am aware of the anatomical areas to avoid while performing this block.
  **Table:**
  Strongly Disagree	Disagree	Neutral	Agree	Strongly Agree
  1	2	3	4	5
6. I am familiar with the contraindications to performing this block.
  **Table:**
  Strongly Disagree	Disagree	Neutral	Agree	Strongly Agree
  1	2	3	4	5
7. I am comfortable choosing an injectable anesthetic for this block.
  **Table:**
  Strongly Disagree	Disagree	Neutral	Agree	Strongly Agree
  1	2	3	4	5
8. I am aware of the potential complications of this block and how to manage them.
  **Table:**
  Strongly Disagree	Disagree	Neutral	Agree	Strongly Agree
  1	2	3	4	5

Please use the below space to free write any concerns or questions you would have performing this block out in the community:

Ultrasound-Guided Hand/Forearm Nerve Blocks Pre-Workshop Questionnaire

1. I am familiar with the indications of the various hand and forearm nerve blocks.
  **Table:**
  Strongly Disagree	Disagree	Neutral	Agree	Strongly Agree
  1	2	3	4	5
2. I am familiar with the anatomical regions that would benefit from anesthetic injection during hand and forearm nerve blocks.
  **Table:**
  Strongly Disagree	Disagree	Neutral	Agree	Strongly Agree
  1	2	3	4	5
3. I am comfortable with probe selection while performing these blocks.
  **Table:**
  Strongly Disagree	Disagree	Neutral	Agree	Strongly Agree
  1	2	3	4	5
4. I am comfortable identifying the anatomical landmarks pertinent to performing these blocks.
  **Table:**
  Strongly Disagree	Disagree	Neutral	Agree	Strongly Agree
  1	2	3	4	5
5. I am aware of the anatomical areas to avoid while performing these blocks.
  **Table:**
  Strongly Disagree	Disagree	Neutral	Agree	Strongly Agree
  1	2	3	4	5
6. I am familiar with the contraindications to performing these blocks.
  **Table:**
  Strongly Disagree	Disagree	Neutral	Agree	Strongly Agree
  1	2	3	4	5
7. I am comfortable choosing an injectable anesthetic for these blocks.
  **Table:**
  Strongly Disagree	Disagree	Neutral	Agree	Strongly Agree
  1	2	3	4	5
8. I am aware of the potential complications of these blocks and how to manage them.
  **Table:**
  Strongly Disagree	Disagree	Neutral	Agree	Strongly Agree
  1	2	3	4	5

Ultrasound-Guided Hand and Forearm Nerve Blocks Post-Workshop Questionnaire

1. I am familiar with the indications of hand and forearm nerve blocks.
  **Table:**
  Strongly Disagree	Disagree	Neutral	Agree	Strongly Agree
  1	2	3	4	5
2. I am familiar with the anatomical regions that would benefit from anesthetic injection during hand and forearm nerve blocks.
  **Table:**
  Strongly Disagree	Disagree	Neutral	Agree	Strongly Agree
  1	2	3	4	5
3. I am comfortable with probe selection while performing these blocks.
  **Table:**
  Strongly Disagree	Disagree	Neutral	Agree	Strongly Agree
  1	2	3	4	5
4. I am comfortable identifying the anatomical landmarks pertinent to performing these blocks.
  **Table:**
  Strongly Disagree	Disagree	Neutral	Agree	Strongly Agree
  1	2	3	4	5
5. I am aware of the anatomical areas to avoid while performing these blocks.
  **Table:**
  Strongly Disagree	Disagree	Neutral	Agree	Strongly Agree
  1	2	3	4	5
6. I am familiar with the contraindications to performing these blocks.
  **Table:**
  Strongly Disagree	Disagree	Neutral	Agree	Strongly Agree
  1	2	3	4	5
7. I am comfortable choosing an injectable anesthetic for these blocks.
  **Table:**
  Strongly Disagree	Disagree	Neutral	Agree	Strongly Agree
  1	2	3	4	5
8. I am aware of the potential complications of these blocks and how to manage them.
  **Table:**
  Strongly Disagree	Disagree	Neutral	Agree	Strongly Agree
  1	2	3	4	5

Please use the below space to free write any concerns or questions you would have performing these blocks out in the community:

Ultrasound-Guided Posterior Tibial Nerve Block Pre-Workshop Questionnaire

1. I am familiar with the indications of a posterior tibial nerve block.
  **Table:**
  Strongly Disagree	Disagree	Neutral	Agree	Strongly Agree
  1	2	3	4	5
2. I am familiar with the anatomical regions that would benefit from anesthetic injection during a posterior tibial nerve block.
  **Table:**
  Strongly Disagree	Disagree	Neutral	Agree	Strongly Agree
  1	2	3	4	5
3. I am comfortable with probe selection while performing this nerve block.
  **Table:**
  Strongly Disagree	Disagree	Neutral	Agree	Strongly Agree
  1	2	3	4	5
4. I am comfortable identifying the anatomical landmarks pertinent to performing this block.
  **Table:**
  Strongly Disagree	Disagree	Neutral	Agree	Strongly Agree
  1	2	3	4	5
5. I am aware of the anatomical areas to avoid while performing this block.
  **Table:**
  Strongly Disagree	Disagree	Neutral	Agree	Strongly Agree
  1	2	3	4	5
6. I am familiar with the contraindications to performing this block.
  **Table:**
  Strongly Disagree	Disagree	Neutral	Agree	Strongly Agree
  1	2	3	4	5
7. I am comfortable choosing an injectable anesthetic for this block.
  **Table:**
  Strongly Disagree	Disagree	Neutral	Agree	Strongly Agree
  1	2	3	4	5
8. I am aware of the potential complications of this block and how to manage them.
  **Table:**
  Strongly Disagree	Disagree	Neutral	Agree	Strongly Agree
  1	2	3	4	5

Ultrasound-Guided Posterior Tibial Nerve Block Post-Workshop Questionnaire

1. I am familiar with the indications of a posterior tibial nerve block.
  **Table:**
  Strongly Disagree	Disagree	Neutral	Agree	Strongly Agree
  1	2	3	4	5
2. I am familiar with the anatomical regions that would benefit from anesthetic injection during a posterior tibial nerve block.
  **Table:**
  Strongly Disagree	Disagree	Neutral	Agree	Strongly Agree
  1	2	3	4	5
3. I am comfortable with probe selection while performing this nerve block.
  **Table:**
  Strongly Disagree	Disagree	Neutral	Agree	Strongly Agree
  1	2	3	4	5
4. I am comfortable identifying the anatomical landmarks pertinent to performing this block.
  **Table:**
  Strongly Disagree	Disagree	Neutral	Agree	Strongly Agree
  1	2	3	4	5
5. I am aware of the anatomical areas to avoid while performing this block.
  **Table:**
  Strongly Disagree	Disagree	Neutral	Agree	Strongly Agree
  1	2	3	4	5
6. I am familiar with the contraindications to performing this block.
  **Table:**
  Strongly Disagree	Disagree	Neutral	Agree	Strongly Agree
  1	2	3	4	5
7. I am comfortable choosing an injectable anesthetic for this block.
  **Table:**
  Strongly Disagree	Disagree	Neutral	Agree	Strongly Agree
  1	2	3	4	5
8. I am aware of the potential complications of this block and how to manage them.
  **Table:**
  Strongly Disagree	Disagree	Neutral	Agree	Strongly Agree
  1	2	3	4	5

Please use the below space to free write any concerns or questions you would have performing this block out in the community:
