# Supplementary figures and images for: Ultrasound Guided Peripheral Nerve Block Workshop: How to Take Your Residents from Zero to Hero
Source: J Educ Teach Emerg Med. 2025 Oct 31;10(4):SG66–86. doi: 10.21980/J8.52156 (PMC12594473; doi:10.21980/J8.52156)

## Slide 1
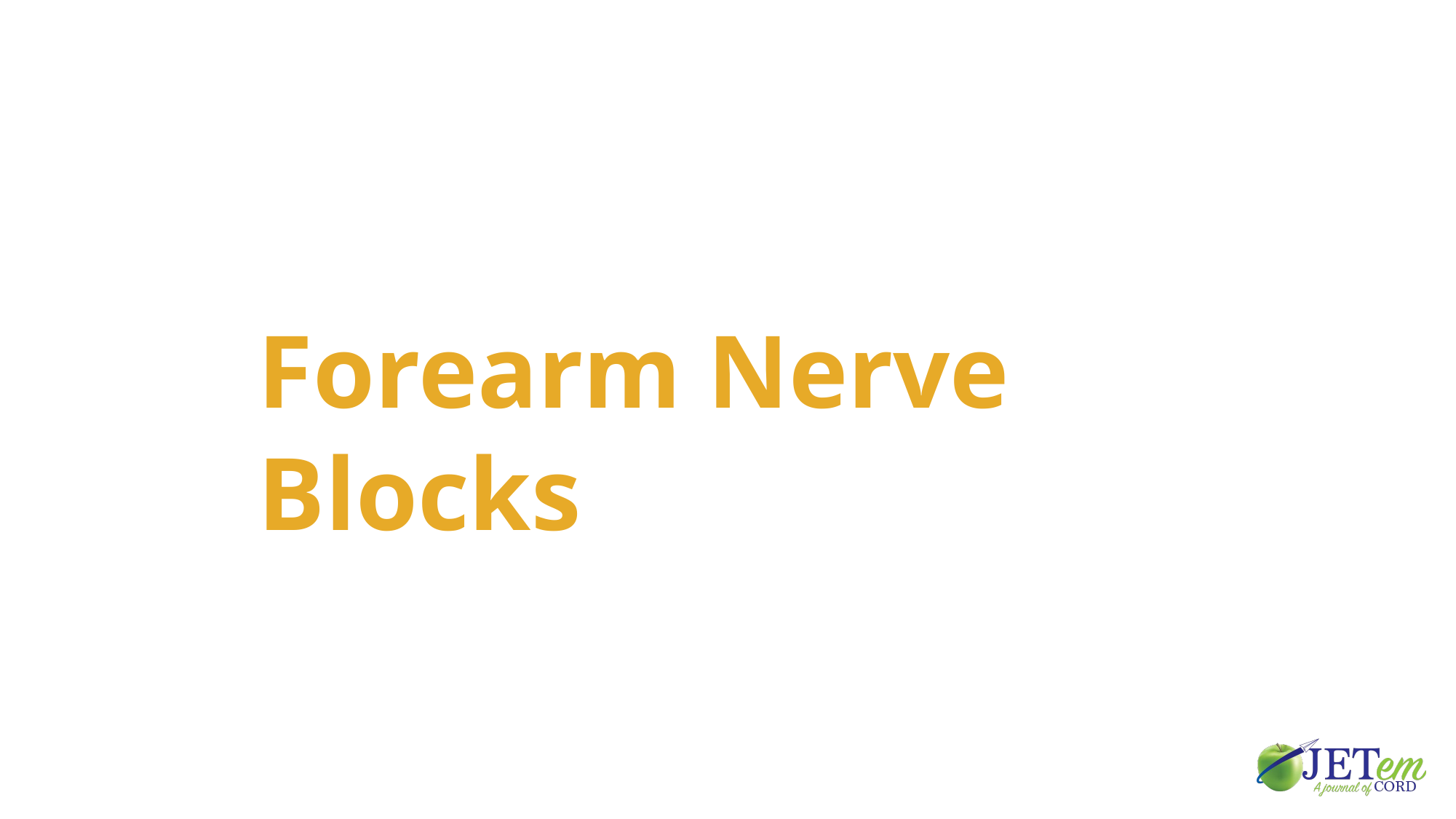

## Slide 2
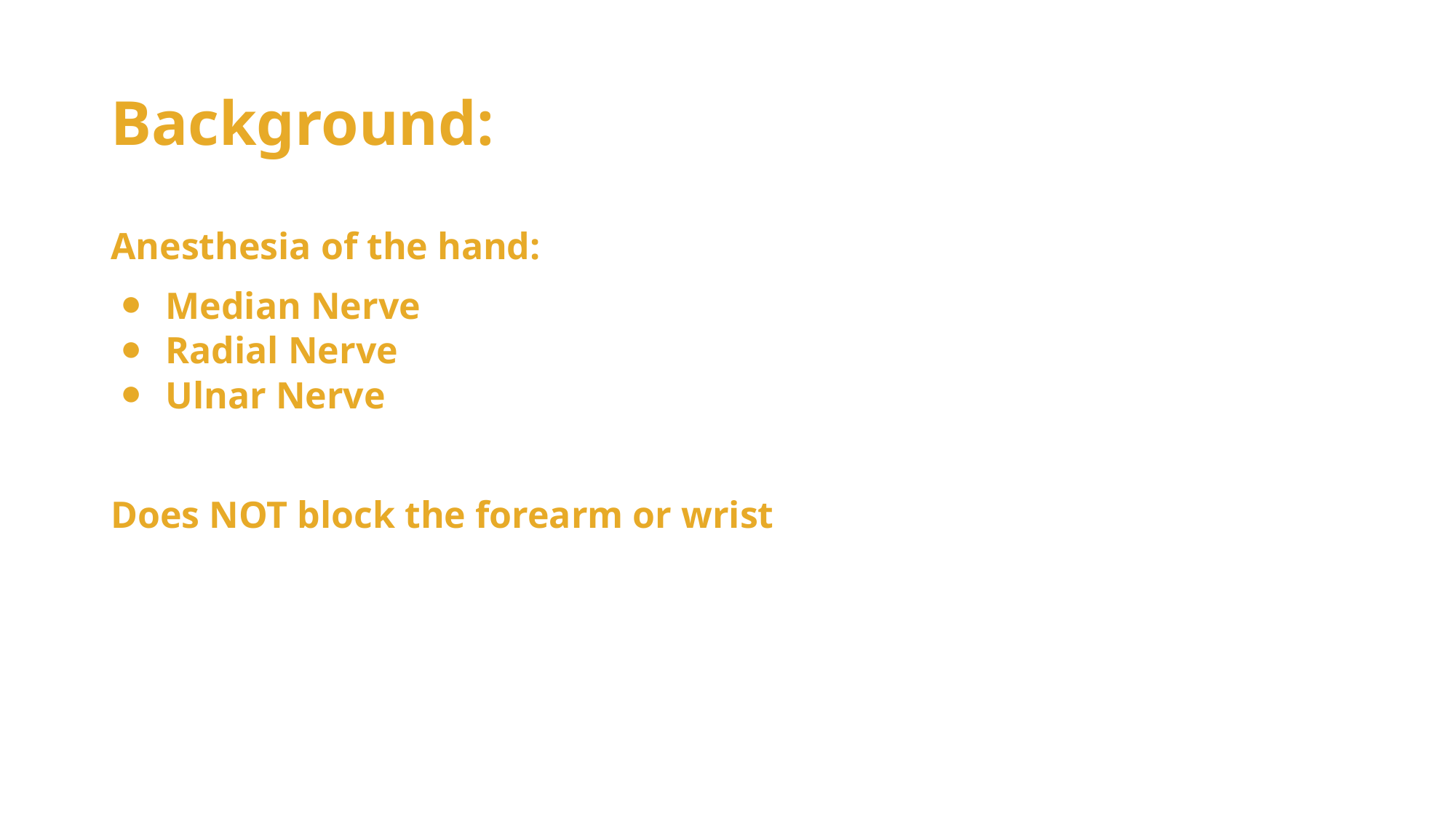

## Slide 3
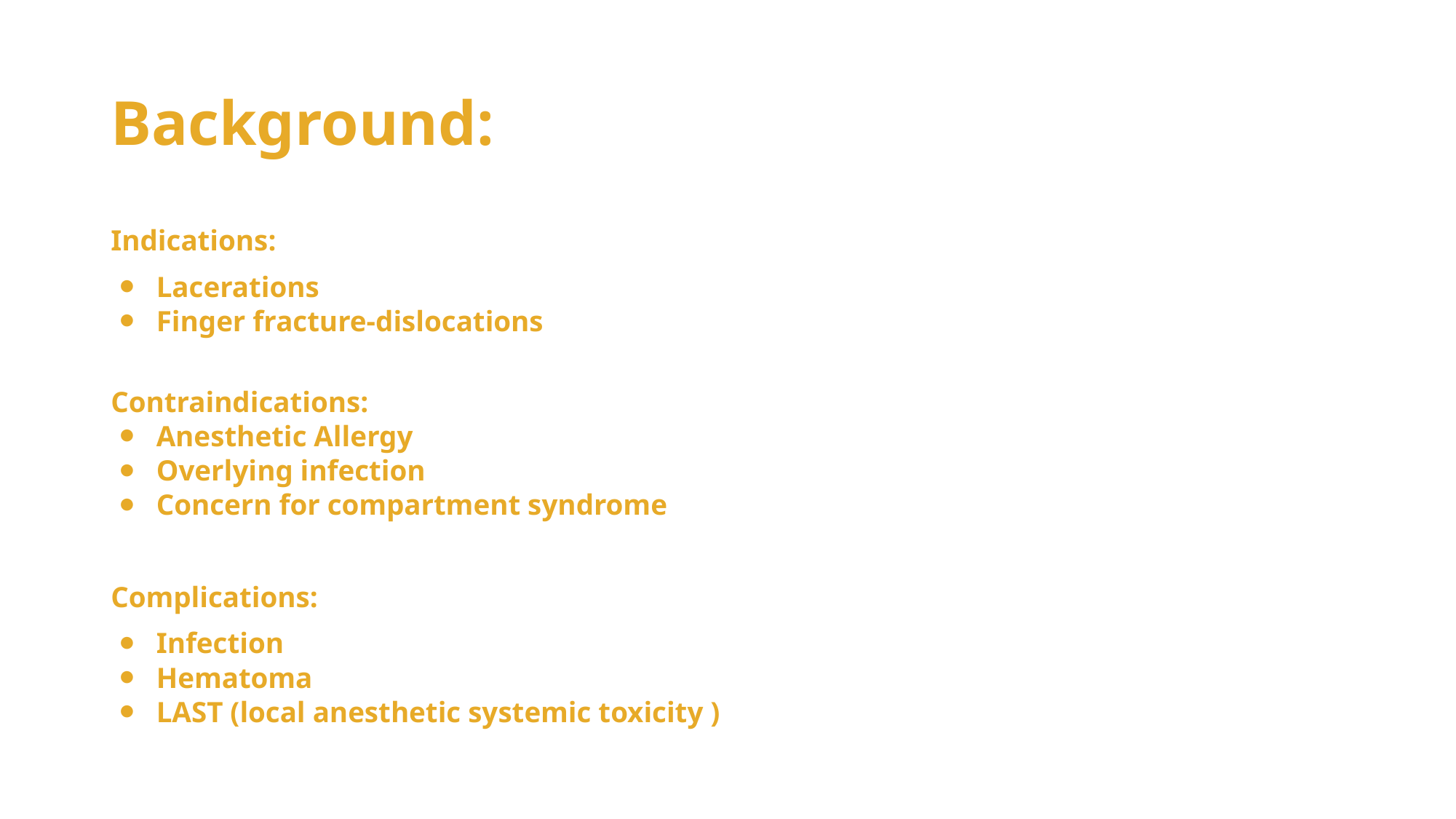

## Slide 4
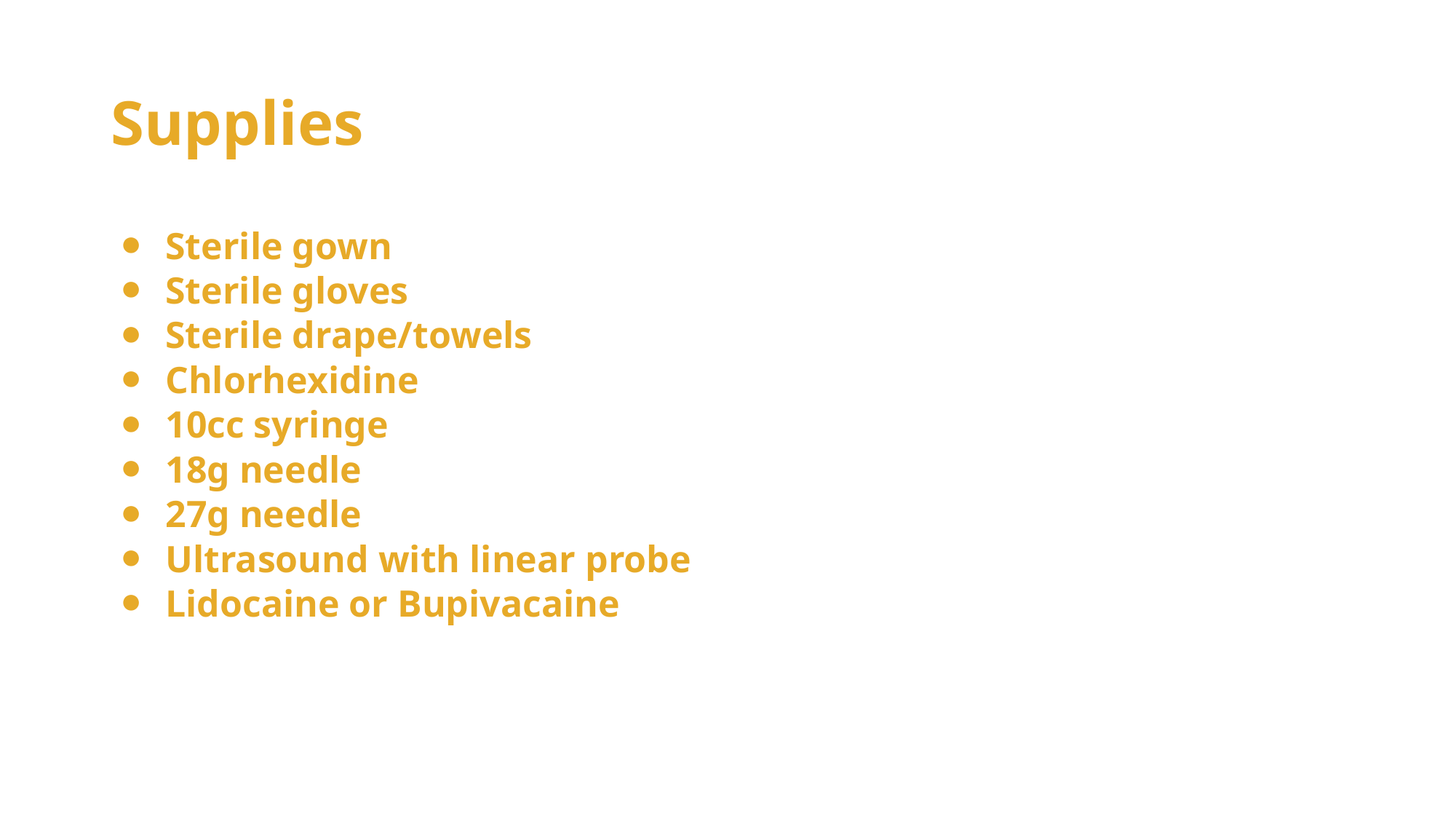

## Slide 5
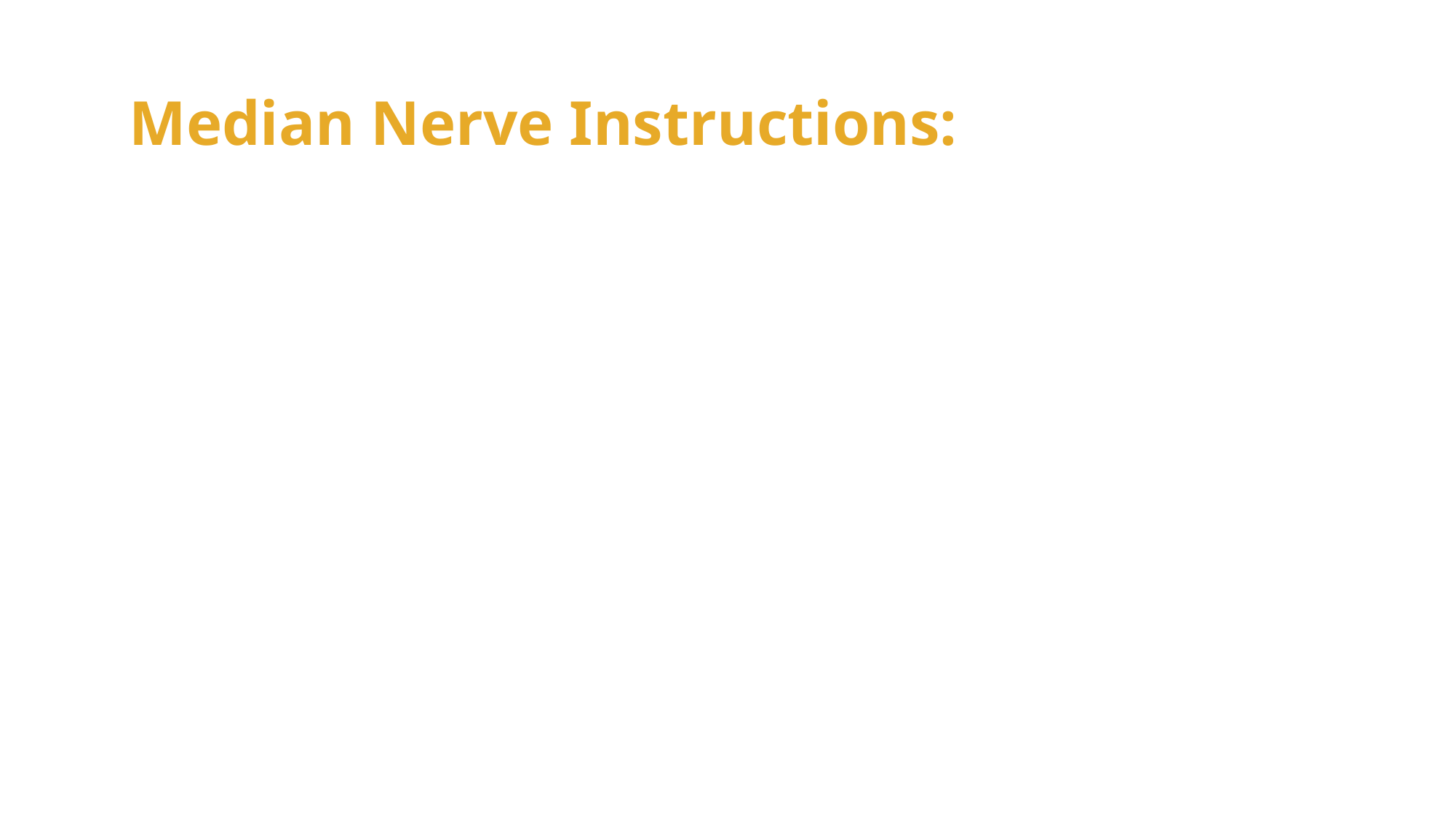

## Slide 6
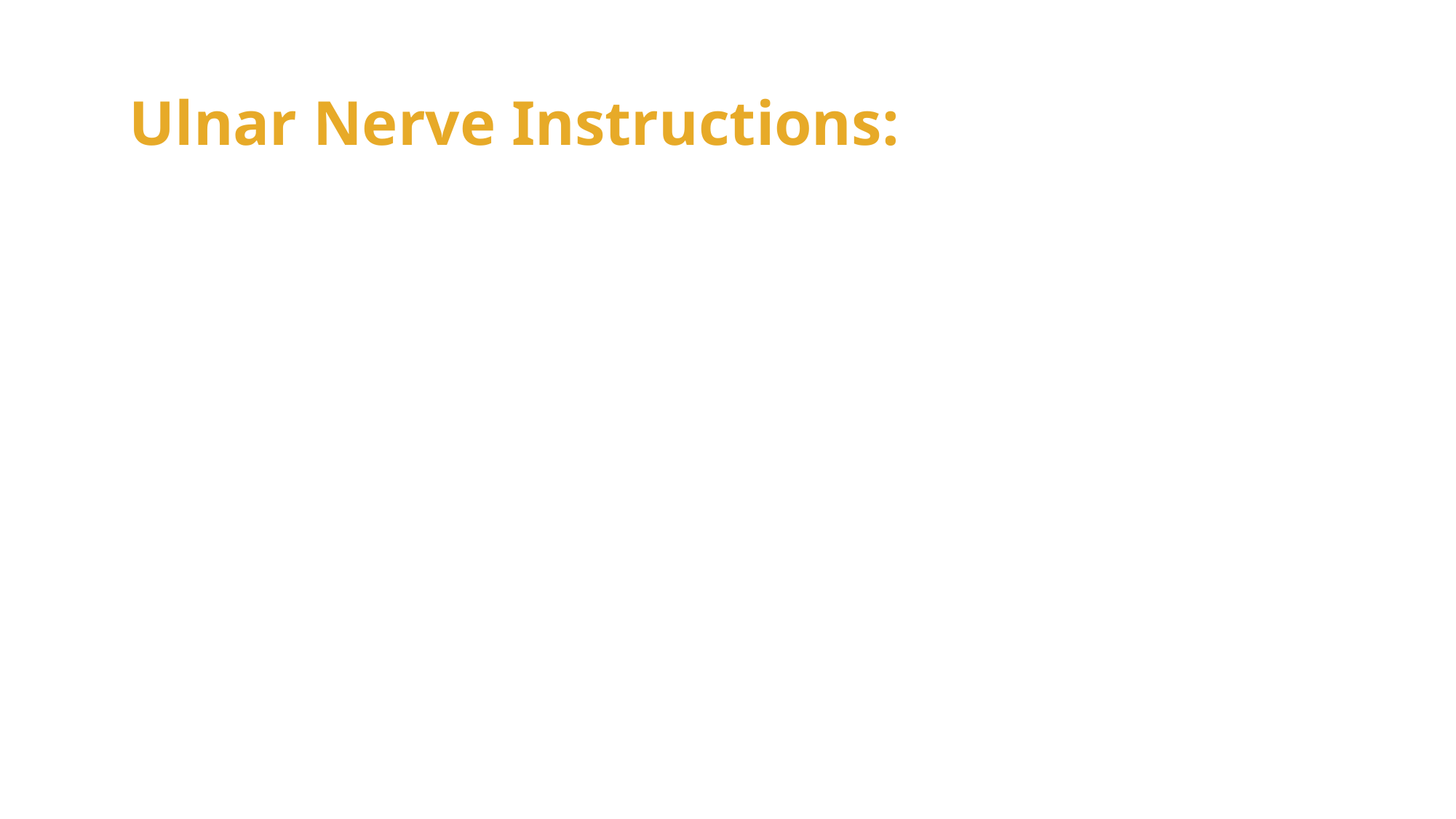

## Slide 7
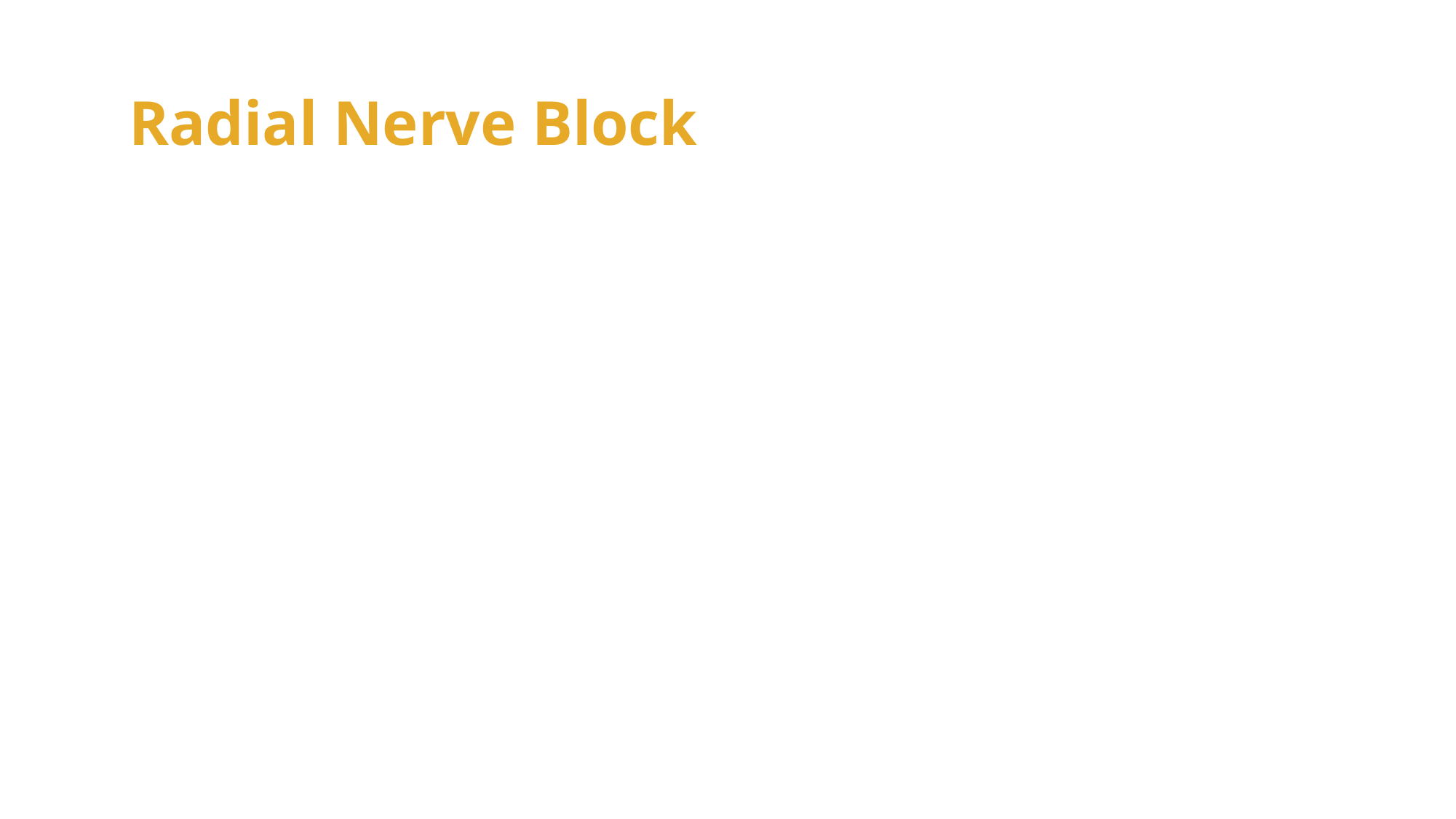

## Slide 8
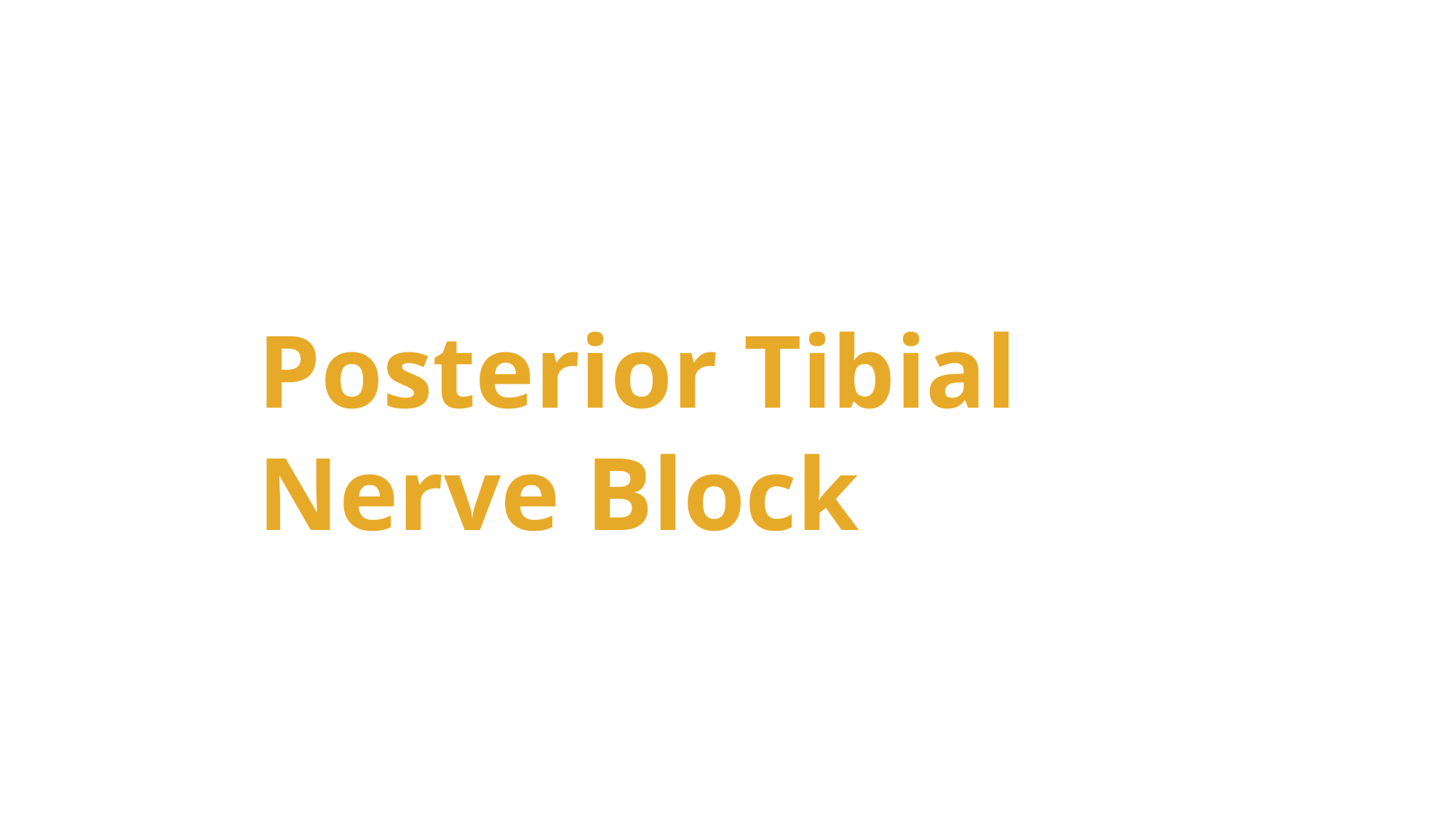

## Slide 9
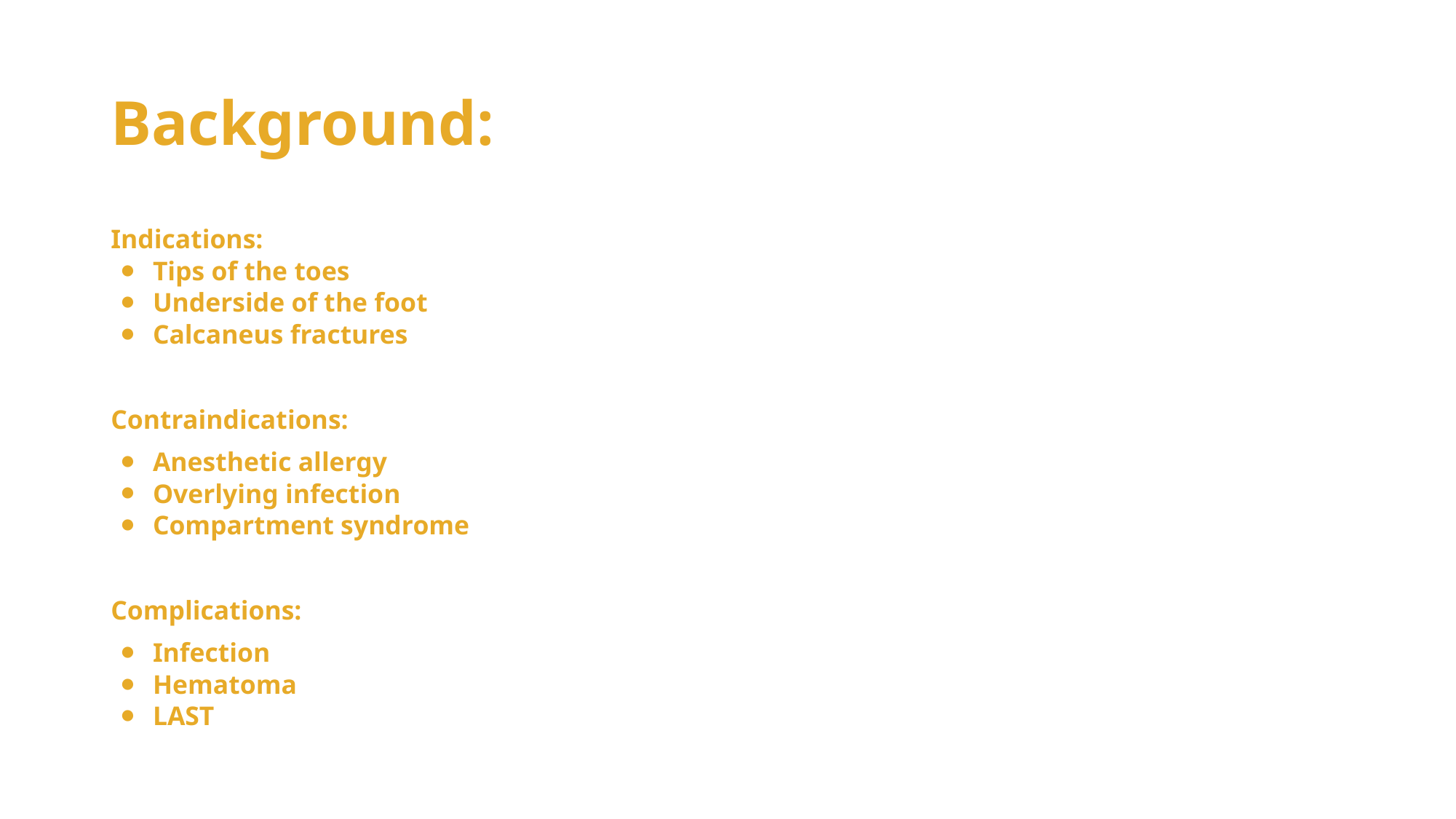

## Slide 10
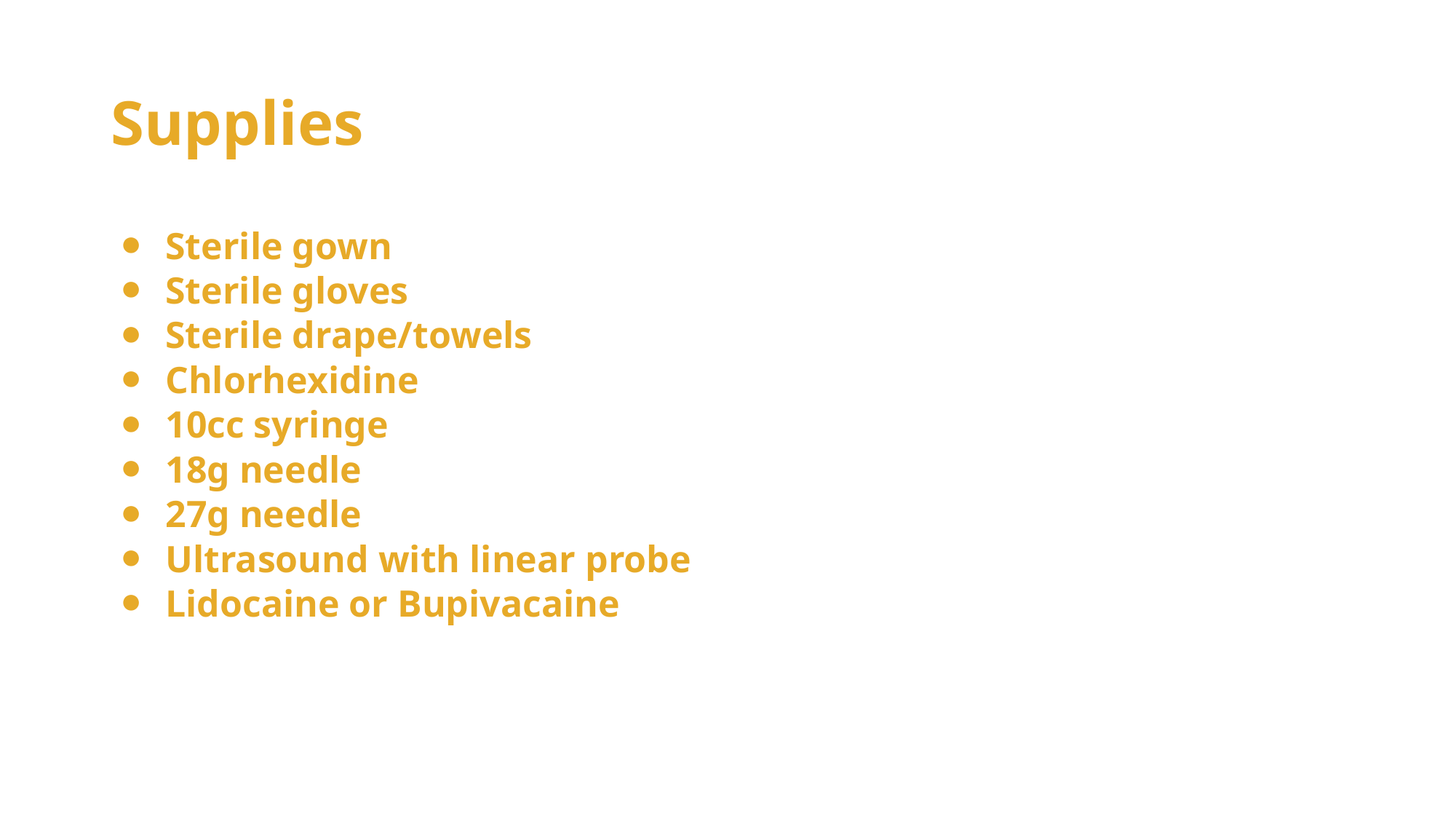

## Slide 11
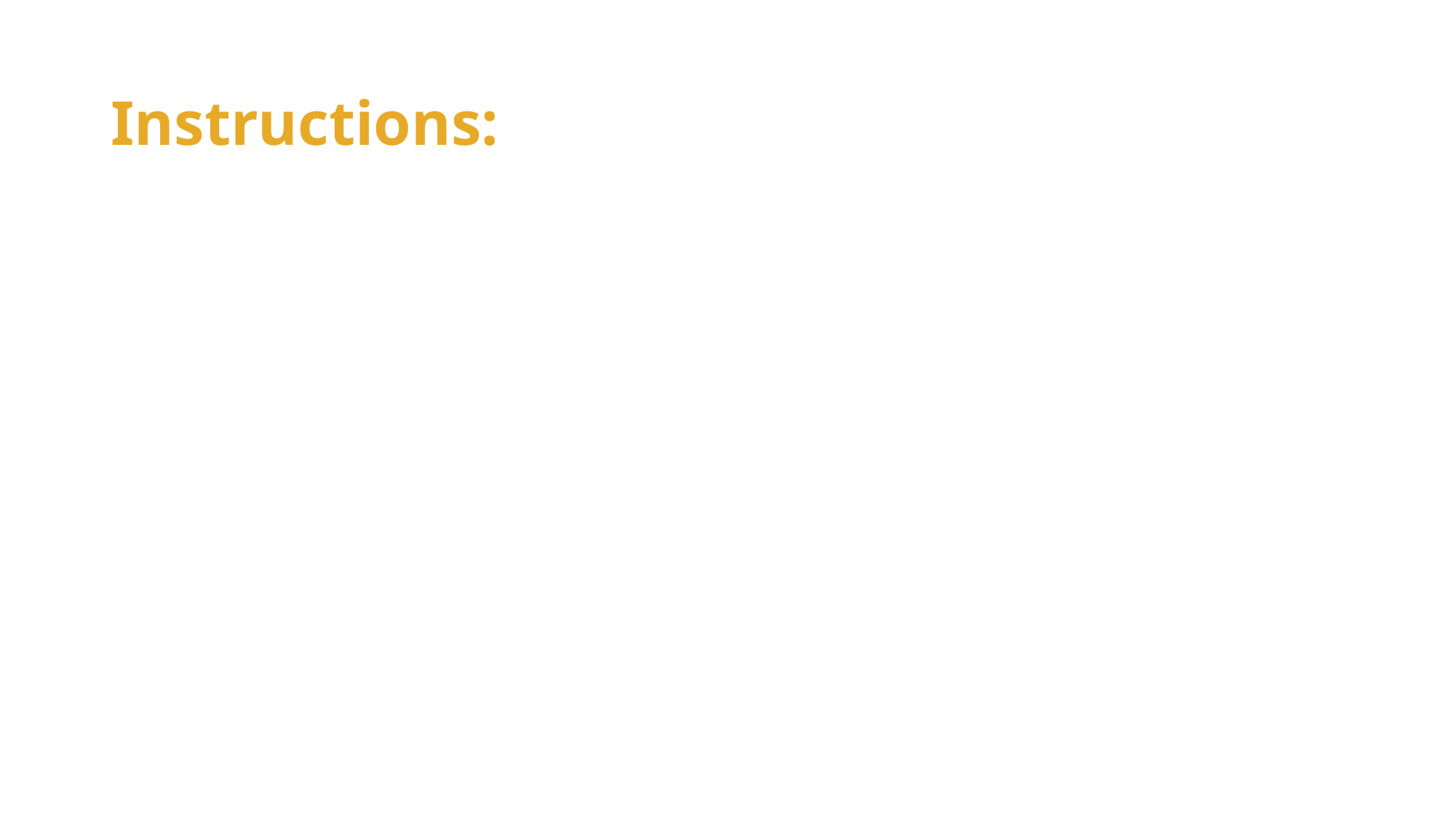

## Slide 12
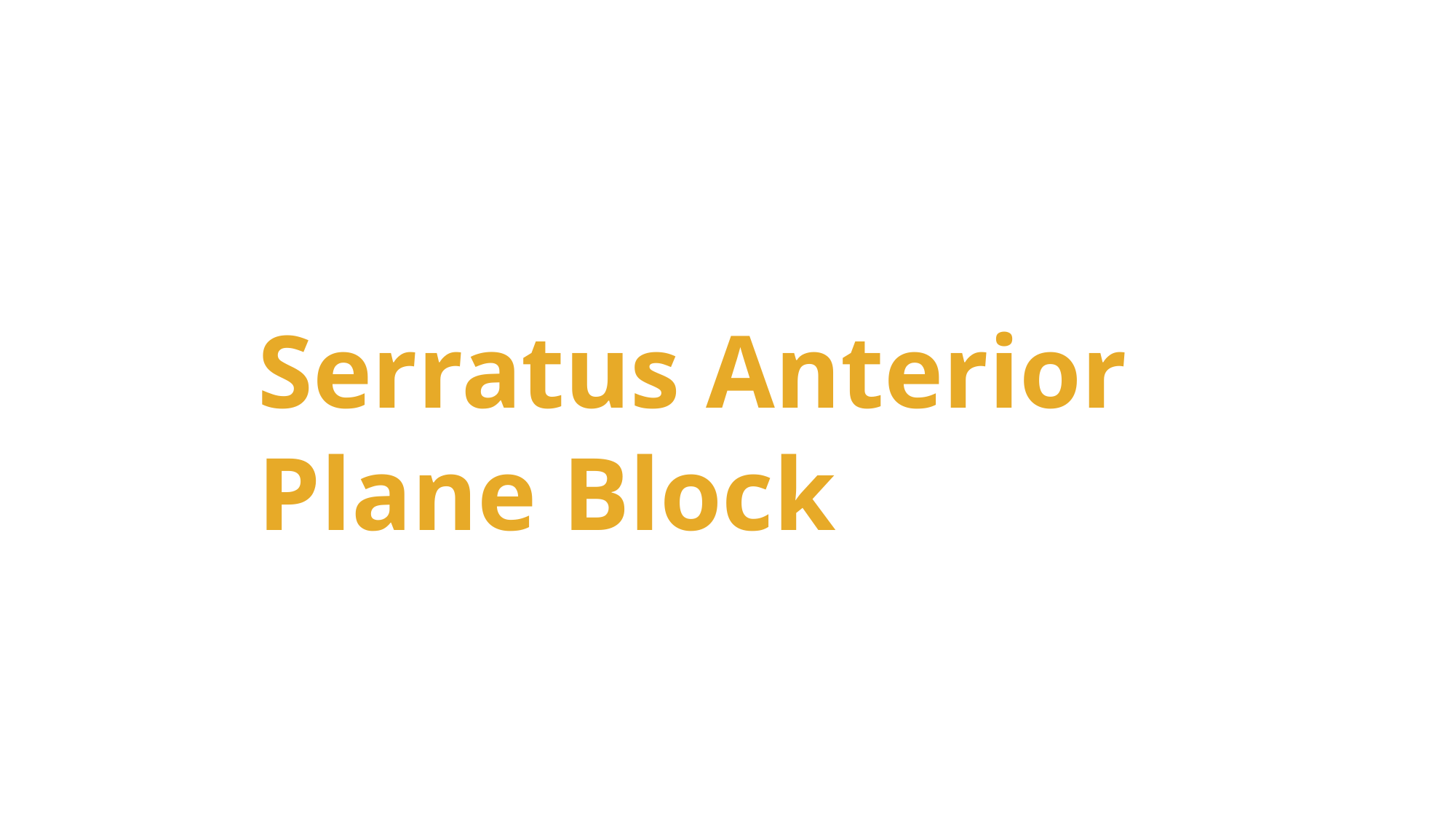

## Slide 13
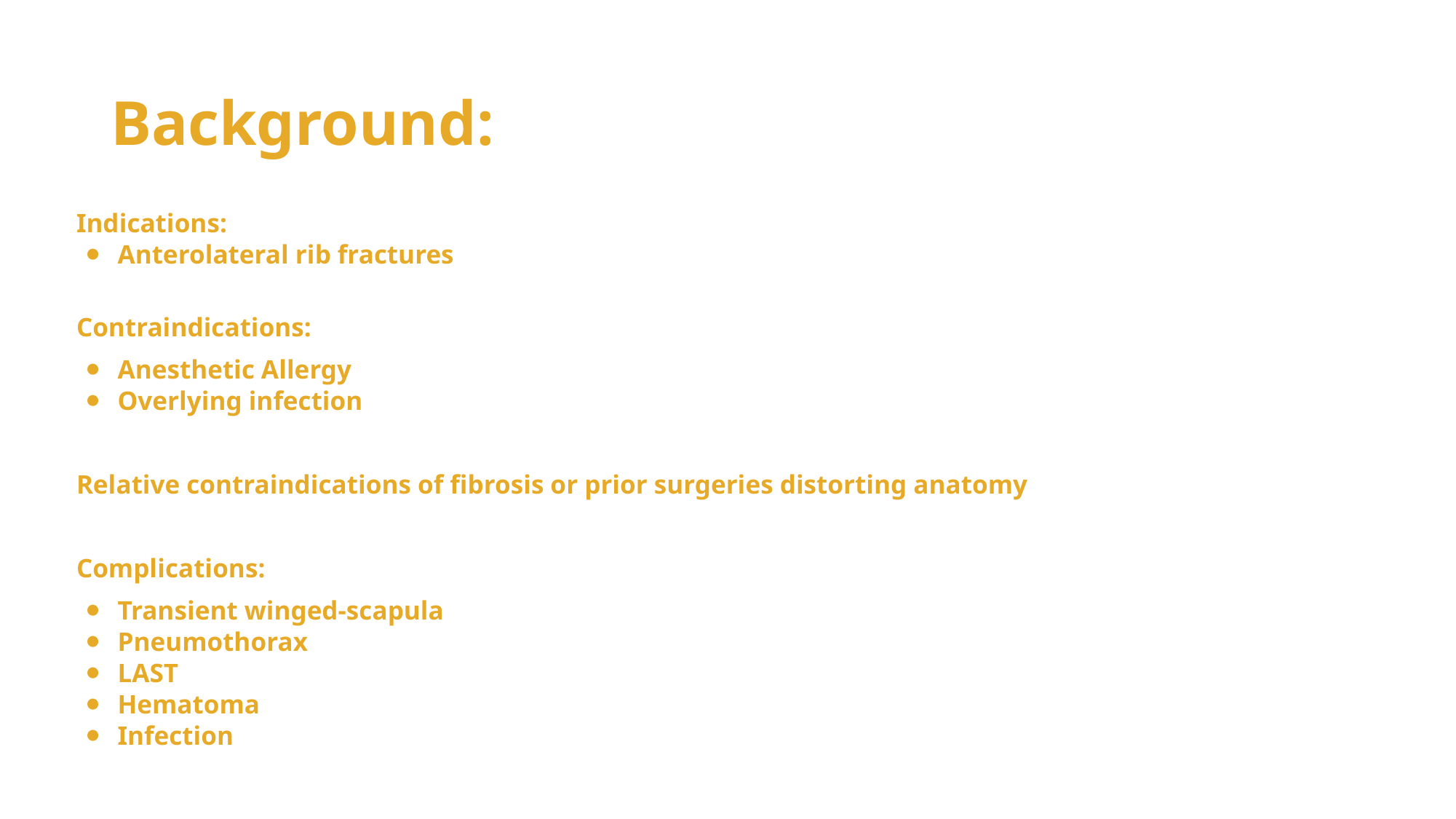

## Slide 14
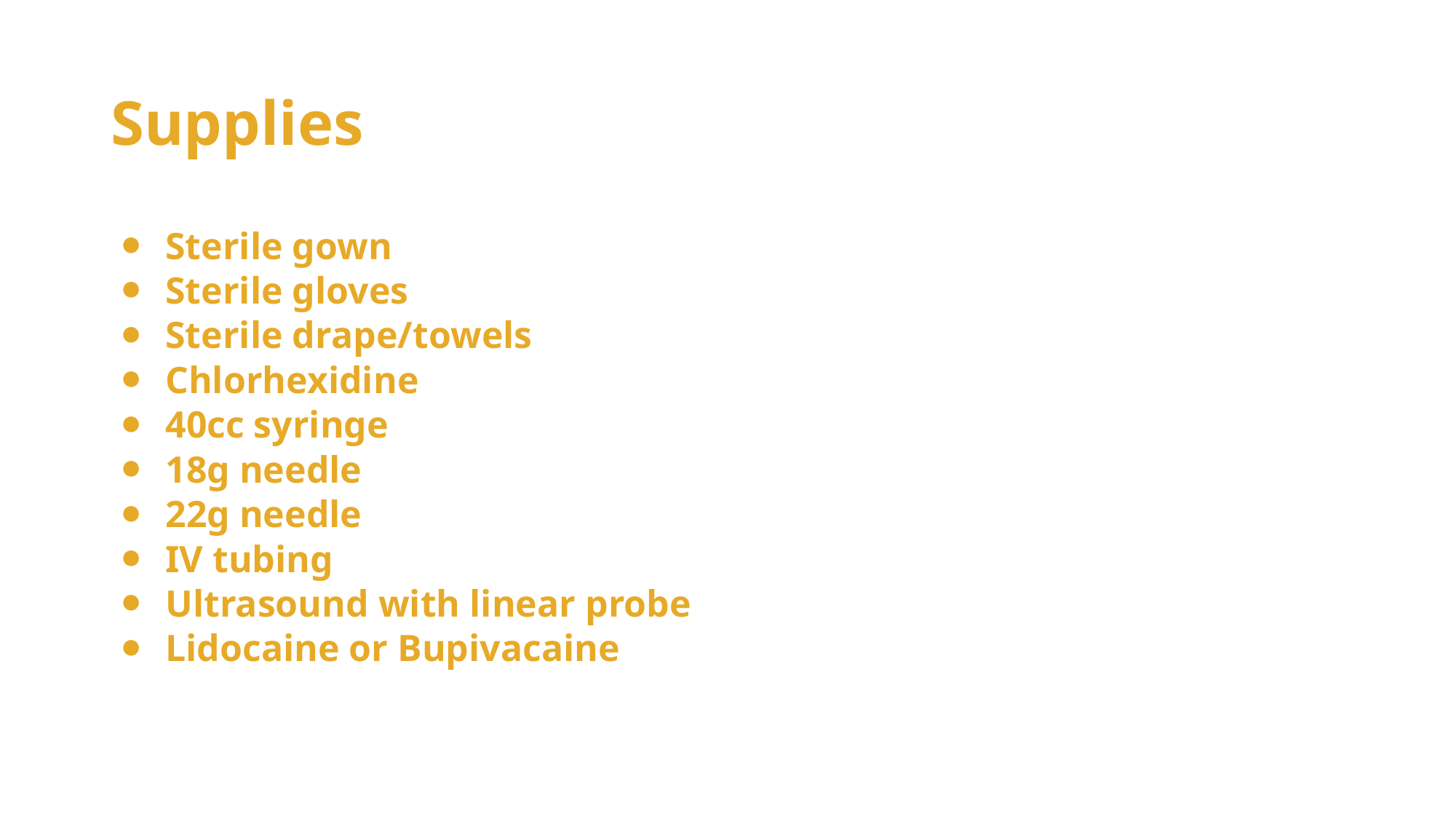

## Slide 15
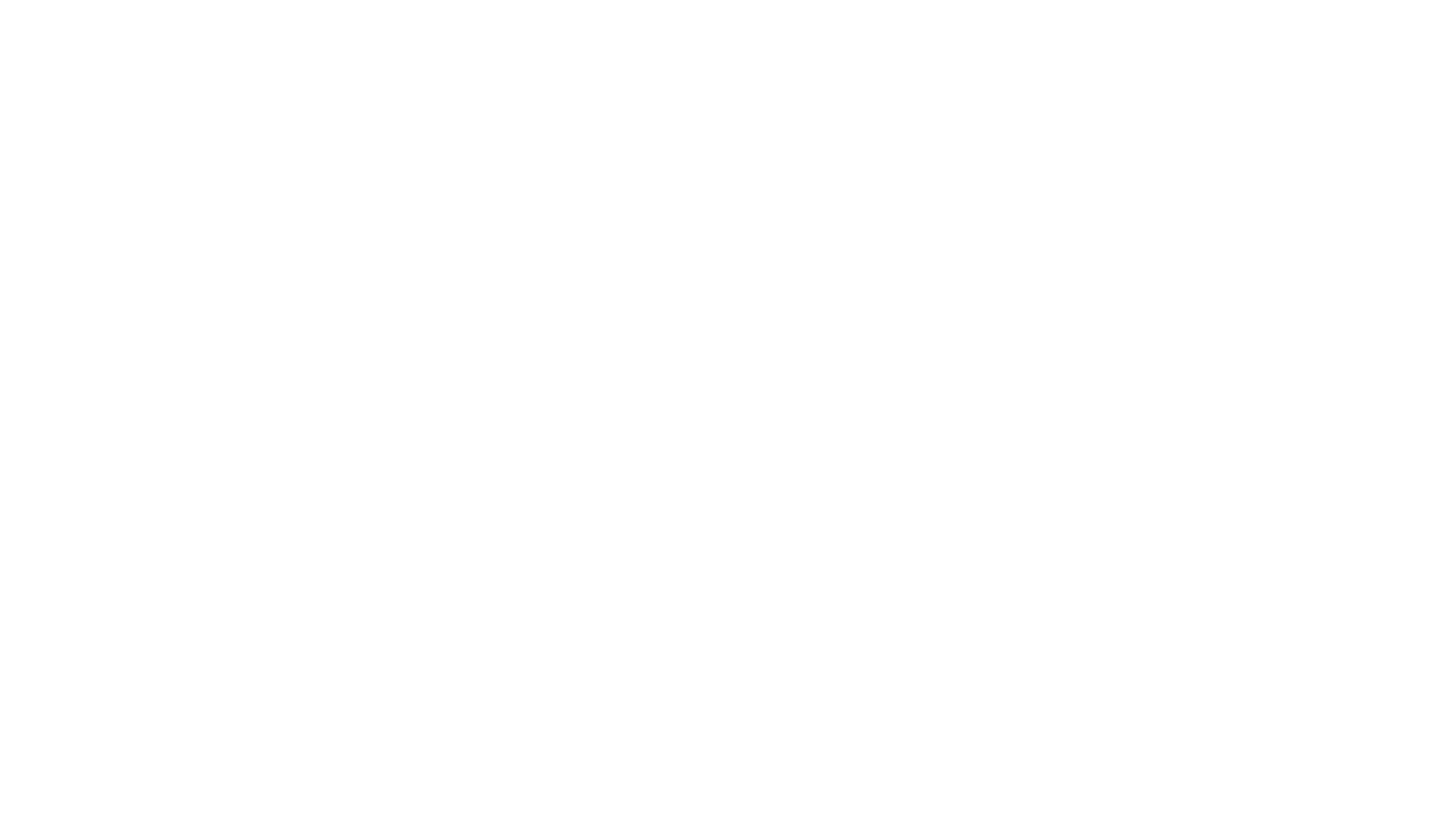

Supplement: Supplementary file 1 [file 10-4-SG66-AppendixA.pptx]
